# Short-Term Inspiratory Muscle Training Enhances Functional and Metabolic Health in Older Adults

**DOI:** 10.3390/healthcare14020249

**Published:** 2026-01-19

**Authors:** Erkan Konca, Coşkun Yılmaz, Serdar Bayrakdaroğlu, Halil İbrahim Ceylan, Ayla Arslan, Hakan Ocak, İzzet Karakulak, Rifat Sarı, Recep Nur Uzun, Hakan Hüseyin Soylu, Levent Ceylan, Raul Ioan Muntean

**Affiliations:** 1 Faculty of Sports Sciences, Sivas Cumhuriyet University, Sivas 58140, Türkiye; erkankonca@cumhuriyet.edu.tr; 2 Kelkit Aydın Doğan Vocational School, Gümüşhane University, Gümüşhane 29600, Türkiye; coskun.yilmaz@gumushane.edu.tr; 3 Faculty of Sports Sciences, Gümüşhane University, Gümüşhane 29100, Türkiye; bayrakdaroglu85@gmail.com; 4 Faculty of Sports Sciences, Atatürk University, Erzurum 25100, Türkiye; 5 Faculty of Medicine, Department of Anatomy, Ağrı Ibrahim Cecen University, Ağrı 04100, Türkiye; ayarslan@agri.edu.tr (A.A.); hocak@agri.edu.tr (H.O.); 6 Faculty of Sport Sciences, Mardin Artuklu University, Mardin 47100, Türkiye; izzetkarakulak@gmail.com; 7 Faculty of Sport Sciences, Tokat Gaziosmanpaşa University, Tokat 62050, Türkiye; rifat.sari@gop.edu.tr; 8 Yasar Doğu Faculty of Sport Sciences, Ondokuz Mayis University, Samsun 55100, Türkiye; recepnur.uzun@omu.edu.tr; 9 Radiology Department, Kelkit District State Hospital, Gumushane Provincial Health Directorate, Gümüşhane 29600, Türkiye; huseyin.soylu.91@gmail.com; 10 Faculty of Sport Sciences, Hitit University, Çorum 19030, Türkiye; leventceylan17@hotmail.com; 11 Faculty of Law and Social Sciences, University “1 Decembrie 1918” of Alba Iulia, 510009 Alba Iulia, Romania

**Keywords:** aging, exercise, fatty liver density, gait, aerobic capacity, health, inspiratory muscle training, elderly men

## Abstract

**Background**: Age-related declines in respiratory muscle strength and ventilatory efficiency can impair functional capacity and metabolic health in older adults. Inspiratory muscle training (IMT) has been proposed as a practical intervention to counteract these changes, yet its systemic effects remain unclear. This study aimed to examine the effects of short-term IMT on functional capacity, diaphragm thickness, and liver tissue characteristics in healthy elderly men. **Methods:** Thirty community-dwelling men aged 60–80 years were randomly assigned to an IMT or control group. The IMT group performed four weeks of breathing exercises using a POWERbreathe^®^ device at 40% of maximal inspiratory pressure, with a weekly 10% increase in pressure. Pre- and post-intervention assessments included the six-minute walk test (6MWT), diaphragm thickness and liver density via computed tomography, and quality of life (QoL; SF-12). **Results:** Four weeks of inspiratory muscle training significantly improved diaphragm thickness (11.7%), fatty liver density (FLD) (+16.7%), and six-minute walk performance (+5.3%), with large time × group effects favoring the IMT group. While the physical quality of life showed modest, comparable improvements, mental health outcomes demonstrated a moderate, time-dependent improvement without a significant group-by-time interaction. **Conclusions:** Short-term IMT improved diaphragmatic function and functional capacity in older men and was associated with favorable changes in a liver-related biomarker; however, given that only a single liver-related metric was assessed, these findings should not be interpreted as evidence of overall improvements in liver health.

## 1. Introduction

Aging is associated with progressive decline in respiratory muscle strength and ventilatory capacity, often resulting in reduced independence and quality of life [1,2]. Age-related increases in abdominal and visceral adiposity can mechanically impair diaphragmatic excursion, reduce chest wall compliance, and contribute to decreased lung volumes and diminished ventilatory efficiency [3]. In men, visceral adiposity is also strongly associated with the development of non-alcoholic fatty liver disease (NAFLD) and broader metabolic dysfunction, further exacerbating cardiometabolic risk [4,5]. Evidence from clinical and athletic populations indicates that inspiratory muscle training (IMT) enhances inspiratory strength, reduces dyspnea, and improves exercise tolerance. Although IMT is well-established for improving inspiratory muscle strength, evidence regarding broader functional or metabolic outcomes in older adults remains limited, with systematic reviews noting inconsistent or modest effects across studies [2,6,7]. The present study addresses this gap by evaluating the combined respiratory, metabolic, and functional effects of a four-week IMT program in older men [8]

Within this framework, physical activity and fitness have emerged as pivotal contributors to overall health and well-being in modern societies. Adequate physical activity is associated with favorable changes in several physiological biomarkers, including reduced systemic inflammation (e.g., lower CRP, IL-6), improved metabolic indicators (e.g., fasting glucose, HbA1c), enhanced cardiorespiratory fitness (VO_2_peak), and healthier liver enzyme profiles (ALT, AST) [9]. This is particularly critical in aging populations, where maintaining physical activity levels supports functional capacity and mitigates age-related physiological decline [10,11]. As aging progresses, health concerns such as sarcopenia, reduced muscle strength, and diminished functional capacity become increasingly prevalent [12].

Respiratory muscle strength declines with aging; however, extensive cohort studies and meta-analyses indicate that maximal inspiratory pressure (MIP) in healthy individuals generally declines by approximately 0.8–2.0 cmH2O per year from the sixth to the seventh decade of life onward. Depending on baseline values, this decline corresponds to roughly 10–25% per decade [13,14]. This reduction in inspiratory muscle strength may contribute to declines in exercise tolerance and habitual physical activity, thereby accelerating overall physiological deterioration with ageing. Although diaphragm thickness has often been proposed as a marker of respiratory muscle status, recent systematic evidence suggests that ultrasonographically measured diaphragm thickness at rest or during contraction may not be a sensitive marker of age-related functional decline in healthy aging. Instead, age-related changes in respiratory muscle function are more consistently reflected in reductions in inspiratory muscle strength, such as MIP, rather than in structural thickness measures [15].

Although many IMT trials in older adults have employed intervention periods of 6–12 weeks, shorter training programs (e.g., ~4 weeks) may still induce early neural and functional adaptations, even if maximal strength gains are generally smaller than those achieved with longer training durations. Accordingly, the present study focuses on the short-term response profile to IMT rather than its maximal long-term effects.

Recent randomized controlled trials and systematic reviews in older adults have strengthened the evidence base for IMT, particularly the randomized controlled studies by Mills et al. [7] and Rodrigues et al. [16], which demonstrated that IMT can elicit meaningful improvements in inspiratory muscle strength, physical performance, and functional capacity in older adults. In addition, a recent systematic review by Seixas et al. [2] further supports the beneficial effects of IMT on respiratory muscle strength and functional outcomes in aging populations. Furthermore, the 2022 European Respiratory Society (ERS) statement provides a comprehensive and contemporary overview of the evidence supporting IMT in older populations.

Nevertheless, evidence from short-term IMT interventions remains heterogeneous. Several randomized controlled trials employing 4–8-week protocols at moderate-to-high intensities (≈50–60% of maximal inspiratory pressure) have reported significant increases in inspiratory muscle strength and, in some cases, clinically meaningful improvements in functional outcomes such as six-minute walk distance and dyspnea [7,16,17]. In contrast, other studies have reported more limited effects on exercise capacity or quality of life. Differences likely influence this variability in baseline inspiratory muscle strength, training intensity and duration, and sample size.

In this context, submaximal exercise assessments, such as the six-minute walk test (6MWT), which more closely reflect daily functional activities, appear particularly suitable for capturing clinically relevant adaptations to short-term IMT. Accordingly, this consideration informed the selection of the 6MWT as a primary outcome in the present study.

The mechanisms underlying IMT are likely multifactorial. The respiratory muscle metaboreflex has been widely described and can influence limb perfusion during periods of high ventilatory work; however, its relative importance appears to vary according to age and exercise context. In older adults, the benefits of short- to medium-term IMT seem to be mediated predominantly by neural adaptations and improved neuromuscular efficiency, along with reductions in perceived exertion and dyspnea, and potentially by structural or metabolic adaptations of the inspiratory muscles (e.g., type I fiber remodeling or enhanced oxidative capacity), rather than solely by attenuation of the metaboreflex [18,19,20]. Consistent with this multimodal framework, inspiratory muscle training has been shown to elicit a range of physiological adaptations, including improvements in respiratory efficiency and ventilatory control, reductions in perceived dyspnea, enhanced exercise endurance, and increases in respiratory muscle strength. Depending on training load and population characteristics, IMT may also promote neuromuscular adaptations of the inspiratory muscles and favorable changes in muscle function and composition [21,22,23]. Collectively, these neural, muscular, and functional adaptations may translate into clinically meaningful improvements in functional performance and health-related quality of life in older adults. Regarding metabolic outcomes, there is currently no evidence for a direct mechanistic link between IMT and hepatic steatosis. Any observed associations should therefore be interpreted cautiously and considered exploratory, as any potential influence is more likely indirect—mediated by factors such as improved activity tolerance or modest changes in daily energy expenditure—rather than a direct physiological effect. As populations continue to age globally, growing international evidence supports IMT as a cost-effective, low-risk intervention to improve respiratory function, functional exercise capacity, and quality of life in older adults.

Based on these rationales, this study hypothesized that IMT would improve diaphragm thickness, fatty liver, quality of life, and six-minute walk test performance in older men. In light of this hypothesis, the objective of this study was to examine the effects of inspiratory muscle training on diaphragm thickness, liver fat percentage, quality of life, and 6 min walk test (6MWT) performance in older men. A comprehensive evaluation of the relationships among these parameters, in conjunction with IMT’s extensive health benefits, may yield critical insights that facilitate the development of novel strategies to preserve functional independence and enhance quality of life in aging populations.

## 2. Materials and Methods

### 2.1. Participants

This study employed a randomized controlled pretest–posttest design in accordance with the CONSORT reporting standards (Supplementary Materials). Before participation, all volunteers were thoroughly informed about the study procedures, and written consent was obtained in line with the ethical guidelines of the Declaration of Helsinki. All participants were informed, both verbally and in writing, prior to enrollment that they could withdraw from the study at any time, without providing a reason and without any consequences for their usual medical care. Ethical approval was granted by the Scientific Research Ethics Committee of Hitit University (Date: 29 August 2025; No: 2025/0464).

The research was designed to evaluate the effects of IMT on diaphragm thickness, abdominal wall thickness, and liver fat density in elderly men aged 60–80 years. Sample size estimation was performed using G*Power (v3.1.9.2) based on diaphragm thickness values from the literature [24], assuming α = 0.05, β = 0.20, and a moderate effect size (f = 0.25). It was calculated that 13 participants per group would be sufficient. To account for potential losses, 15 participants were recruited per group, exceeding the minimum required sample size, for a total of 30 participants. Participants were randomly assigned to the IMT (n = 15) and control group (n = 15) using a computer-generated randomization tool (https://www.randomizer.org/).

The IMT group underwent four weeks of respiratory muscle training. In contrast, the control group continued their usual medical care and did not receive inspiratory muscle training, respiratory physiotherapy, or structured exercise interventions. Supervised sessions were conducted by a trainer certified in exercise science with specific training in respiratory muscle training. Participants in the control group received no additional lifestyle counseling, exercise recommendations, or respiratory advice beyond routine clinical follow-up. Participants consistently performed training sessions at the same time of day (morning or evening) throughout the intervention period and did not alternate between time slots. They were only monitored during baseline and follow-up assessments. Inclusion criteria required participants to be nonsmokers, functionally independent, cognitively capable of following instructions, and free from musculoskeletal injuries in the past six months. Given the strong influence of body composition, hydration status, nutritional status, and habitual physical activity on respiratory, neuromuscular, and metabolic outcomes in older adults, the present study aimed to minimize baseline heterogeneity through strict eligibility criteria. Accordingly, only community-dwelling, functionally independent, clinically stable older men without diagnosed metabolic, hepatic, or inflammatory diseases were included. Exclusion criteria encompassed uncontrolled cardiovascular or pulmonary disorders, recent respiratory infections, chronic liver disease, hypertension, or the use of drugs that might alter muscle or liver metabolism. Individuals engaged in any structured exercise or respiratory training within the previous six months were also excluded. Participants were excluded if they were using medications known to substantially influence skeletal muscle or liver metabolism, including systemic corticosteroids, anabolic-anti-catabolic agents, hepatotoxic drugs (e.g., high-dose methotrexate), lipid-lowering therapies (e.g., statins), or thyroid hormone replacement at non-stable doses. Moreover, participants with conditions known to substantially alter muscle function, hydration balance, or metabolic regulation—including chronic liver disease, uncontrolled cardiopulmonary disorders, recent infections, or medications affecting muscle or liver metabolism—were excluded. This approach was intended to ensure that the observed outcomes primarily reflected baseline physiological status and short-term training responses, rather than confounding effects related to disease, malnutrition, or dehydration.

### 2.2. Experimental Design

**Figure 1:**
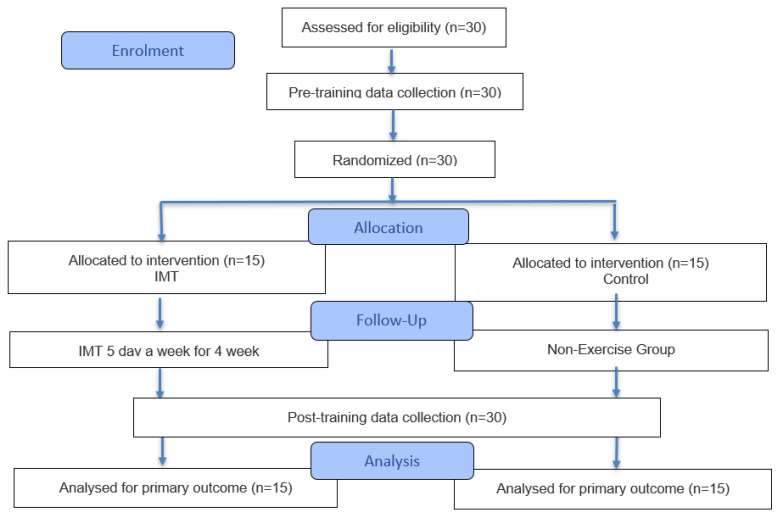
Experimental design.

Each participant attended the laboratory on three separate occasions. During the initial session, the study objectives and testing protocols were introduced, and the IMT device was demonstrated. The second session included baseline assessments: diaphragm muscle thickness (DT), fatty liver density (FLD), the six-minute walk test (6MWT), and quality of life (SF-12). Following four weeks of training, post-intervention evaluations were performed using the same procedures and measurement conditions. Figure 1 illustrates the study flow. The habitual level of physical activity at the start of the study was assessed using the International Physical Activity Questionnaire (short form), not to evaluate changes related to the intervention, but to determine participants’ baseline activity profiles and reduce inter-individual variability at the start of the study.

### 2.3. Assessment of Fatty Liver Density (Fld)

Liver fat density was determined via computed tomography (CT) imaging using a Siemens Somatom Definition AS 128 scanner. Imaging was conducted in the supine position following standardized breathing instructions, during which participants inhaled deeply and held their breath at full inspiration. Images were obtained at the liver dome level, and regions of interest (ROIs) were selected from homogeneous parenchymal areas near segment VIII, adjacent to the middle hepatic vein. The mean of three repeated measurements was recorded as the final value [25]. Hepatic attenuation was assessed using a single ROI in segment VIII on non-contrast CT. This approach was selected due to data availability and feasibility constraints, and findings are therefore interpreted as exploratory rather than diagnostic. The method does not reflect current gold-standard techniques (e.g., MRI-PDFF or CAP). Liver fat content was estimated based on tissue attenuation; values below 33 Hounsfield units were considered indicative of higher hepatic fat infiltration. A threshold of <33 HU in a single ROI was used depending on data availability; however, it is not accepted as a valid diagnostic method for hepatic steatosis under current standards. Therefore, all liver fat analyses were considered exploratory [26]. The same radiologist performed all measurements to ensure consistency, and the intraclass correlation coefficient (ICC) for FLD reliability was 0.844, reflecting good agreement.

### 2.4. Evaluation of Diaphragm Thickness (Dt)

Diaphragm muscle thickness was analyzed using CT scans obtained for clinical purposes with a GE Revolution EVO Cardiac system (GE Healthcare, Waukesha, WI, USA). Measurements were performed by an experienced radiologist during the inspiratory phase, with participants lying supine. Diaphragm thickness was measured from a routine non-contrast CT slice at the level of the upper pole of the right kidney. This non-standard approach was used due to data availability and should be interpreted as exploratory rather than as equivalent to validated B-mode ultrasound methods. Each image was measured three times, and the average value was used for analysis. The ICC for diaphragm thickness reproducibility was calculated as 0.823, indicating good reliability.

### 2.5. Anthropometric and Body Composition Measurements

Body height and weight were recorded using standardized equipment (Seca 769 stadiometer; Seca GmbH, Hamburg, Germany, and Beurer GS27 digital scale; Beurer GmbH, Ulm, Germany). Height was measured to the nearest 0.1 cm, and body weight to the nearest 0.1 kg. Participants were measured barefoot and wearing lightweight clothing. Body mass index (BMI) was calculated as body weight divided by height squared (kg/m^2^). Although detailed body composition analyses were not performed, BMI was used as a pragmatic indicator of general body mass status at baseline. Although BMI does not capture body composition in detail, the relatively narrow BMI range and the absence of underweight or severely obese individuals suggest a reasonably homogeneous baseline body mass profile in the present cohort. Participants with extreme body weight profiles were not represented in the study cohort, as evidenced by relatively homogeneous anthropometric characteristics across groups.

### 2.6. International Physical Activity Questionnaire (IPAQ)

In this study, physical activity levels were determined using the Turkish version of the International Physical Activity Questionnaire (short form) developed by Craig et al. (2003) [27]. The Turkish adaptation study conducted by Öztürk (2005) indicated that the scale is valid and reliable [28]. The questionnaire includes questions about physical activities lasting ≥10 min performed during the past week (how many days and how many minutes per week) [27]. The total physical activity score was calculated in MET-minutes/week using the recommended formulas for each participant. The physical activity score obtained was classified as low (<600 MET-min/week), moderate (601–3000 MET-min/week), and high (>3000 MET-min/week) [28,29].

### 2.7. Quality of Life Assessment (Qol)

Quality of life was evaluated using the validated Turkish version of the Short Form-12 (SF-12) questionnaire [30], which includes physical (PCS) and mental (MCS) component summaries derived from eight health domains [31]. Scores range from 0 to 100, with higher values indicating better perceived health. Internal consistency reliability was confirmed in the present study (Cronbach’s α = 0.737 for PCS and 0.729 for MCS). Scoring was computed using the online calculator available at https://orthopowertools.com/SF12 (accessed on 1 November 2025).

### 2.8. Inspiratory Muscle Training Protocol

The inspiratory muscle training intervention utilized a POWERbreathe^®^ Classic device (IMT Technologies Ltd., Birmingham, UK). Training sessions were conducted twice daily, five days per week, for four consecutive weeks. Each session comprised 30 inspiratory efforts, yielding 60 repetitions per day. Training intensity was initially set at 40% of each participant’s maximal inspiratory pressure (MIP) and was increased weekly by 10% [32]. IMT sessions were performed at home five days per week. Supervision occurred once per week in the laboratory/clinic, during which a certified instructor assessed technique, intensity, and adherence. The remaining home sessions were unsupervised but monitored via training logs. Morning sessions occurred between 08:00 and 10:00, and evening sessions between 17:00 and 20:00. A lower-intensity, short-duration IMT protocol (40–50% MIP, 2 × 30 breaths/day for 4 weeks) was selected to enhance feasibility and adherence in older adults, with the intention of examining early-phase physiological adaptations rather than maximal training responses.

### 2.9. Statistical Analysis

Data analysis was performed using SPSS (v27.0, IBM Corp., Chicago, IL, USA). Normality of data distribution was tested using the Shapiro–Wilk method, and homogeneity of variances was confirmed using Levene’s test (*p* > 0.05). A two-way repeated-measures ANOVA with Bonferroni post hoc tests was used to analyze time-by-group interaction for DT, FLD, 6MWT, and SF-12 outcomes. Statistical significance was accepted at *p* < 0.05. Effect sizes for ANOVA were reported as partial eta squared (ηp^2^), calculated as SS_effect/(SS_effect + SS_error), and interpreted as small (0.01), medium (0.06), or large (≥0.14) [33]. Measurement reliability was assessed using the intraclass correlation coefficient (ICC), with values >0.75 indicating good and >0.90 excellent reproducibility [34].

## 3. Results

A total of thirty older men completed the intervention without any adverse events. Age, body weight, height, BMI, and total physical activity levels are shown in Table 1. Participants with extreme or heterogeneous body composition profiles were excluded to ensure homogeneous anthropometric characteristics across groups.

**Figure 2:**
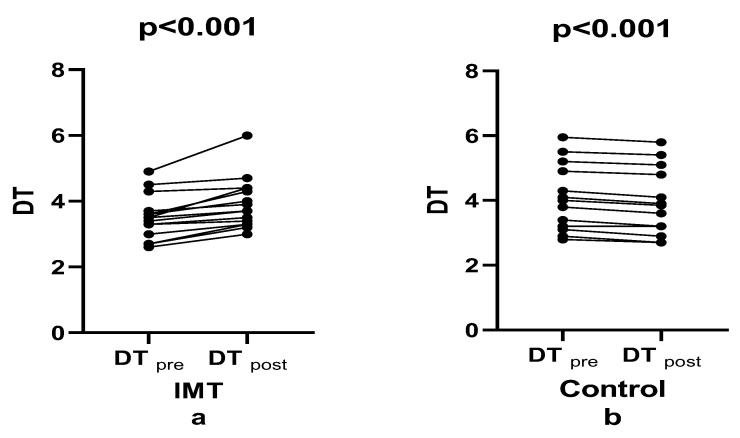
Comparison of diaphragm thickness between groups pre- and post-training.

Figure 2 shows that after the four-week intervention, the mean diaphragm thickness between the pre-test and post-test significantly increased in the IMT group (e.s.: 0.572), while a slight decrease was observed in the control group (e.s.: 0.155) (F(1, 28) = 11.007, *p* = 0.003, np^2^: 0.282; Figure 2). Bonferroni-adjusted analyses revealed a statistically significant increase in mean diaphragm thickness from pre-test to post-test (*p* < 0.003). The IMT group showed an increase of 11.68%, while the control group showed a decrease of 3.65%, resulting in a net improvement of 15.33%. This change produced a large effect size in the interaction effect between groups and test times (F(1, 28) = 52.287, *p* < 0.001, ηp^2^ = 0.651, *p* < 0.001). These results indicate that short-term IMT effectively prevented age-related decreases in diaphragm muscle thickness.

**Figure 3:**
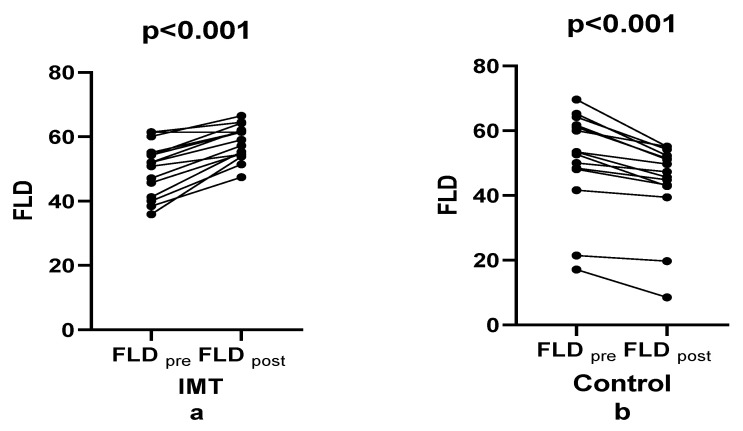
Comparison of fatty liver density between groups pre- and post-training.

Figure 3 illustrates that, following the four-week intervention, mean FLD values increased significantly from pre-test to post-test in the IMT group (effect size: 1.188), whereas the control group showed a slight decrease (effect size: 0.506). Bonferroni-adjusted between-group analyses revealed no significant main effect of time on the dependent variable (F(1, 28) = 0.57, *p* = 0.458, ηp^2^ = 0.020), indicating that overall temporal changes were not significant when groups were considered collectively. While the IMT group exhibited a 16.68% increase, the control group showed a 14% decrease, yielding a net improvement of 30.683%. Consequently, a statistically significant time × group interaction was observed (F(1, 28) = 100.22, *p* < 0.001, ηp^2^ = 0.782), indicating that the pattern of change over time differed markedly between groups. The large effect size indicates a substantial difference between groups across measurement time points.

**Figure 4:**
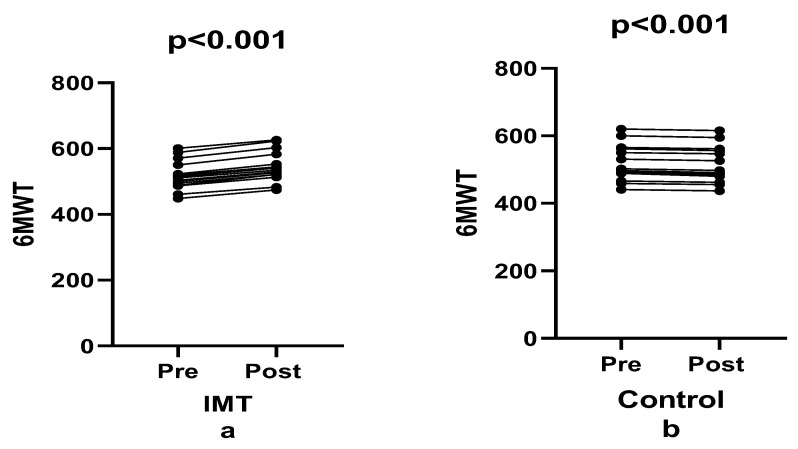
Comparison of 6MWT between groups pre- and post-training.

Performance in the six-minute walk test improved significantly following IMT (F(1, 28) = 443.44, *p* < 0.001, ηp^2^ = 0.941; Figure 4). The IMT group demonstrated a 5.34% increase in walking distance (effect size: 0.618), whereas the control group showed a 0.89% decrease (effect size: 0.089), resulting in a between-group difference of 6.23% (*p* < 0.001). Consequently, a statistically significant time × group interaction was detected (F(1, 28) = 873.65, *p* < 0.001, ηp^2^ = 0.969), indicating that both the magnitude and direction of temporal changes differed markedly between groups. The exceptionally large effect sizes indicate a strong, differential response to the intervention, demonstrating a significant improvement in functional walking performance among participants who underwent inspiratory muscle training.

**Figure 5:**
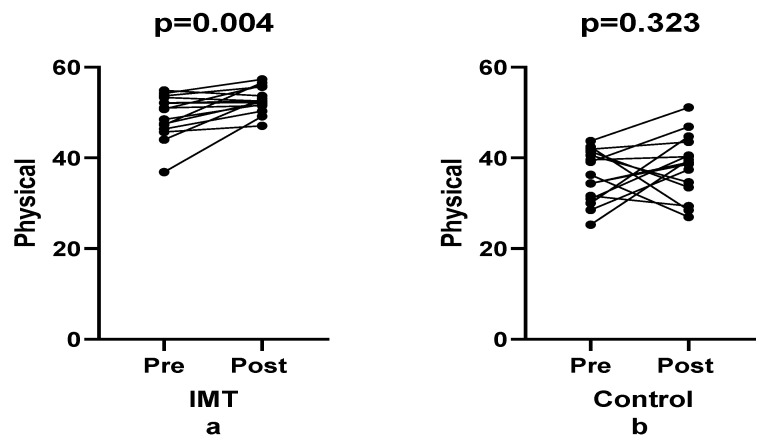
Comparison of physical sub-dimension between groups pre- and post-training.

The physical health subscale of the SF-12 demonstrated a modest improvement in both groups following the intervention. The IMT group exhibited a 7.23% increase (effect size: 0.907), while the control group showed a 6.38% improvement (effect size: 0.363), resulting in a significant main effect of time (F(1, 28) = 5.63, *p* = 0.025, ηp^2^ = 0.167, Figure 5). In contrast, no significant time × group interaction was observed (F(1, 28) = 0.26, *p* = 0.613, ηp^2^ = 0.009), indicating that the pattern of change over time was comparable between groups. The small interaction effect size further suggests minimal differential responsiveness between groups across time. Collectively, these findings indicate that IMT did not exert a substantial influence on the physical component of health-related quality of life over the short intervention period.

**Figure 6:**
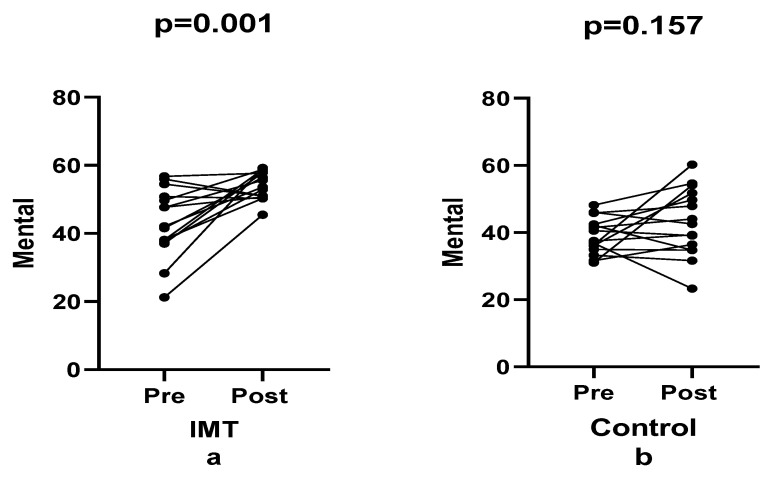
Comparison of mental sub-dimension between groups pre- and post-training.

In contrast, the mental health subscale exhibited a more pronounced change. The IMT group demonstrated a 25.06% improvement, compared with a 10.27% increase in the control group, resulting in a between-group difference of 14.79%. Although this difference did not reach statistical significance, a significant main effect of time was observed (F(1, 28) = 15.00, *p* < 0.001, ηp^2^ = 0.349; Figure 6), accompanied by a moderate effect size. These findings suggest a moderate improvement in perceived mental health in the IMT group relative to the control group. However, the time × group interaction did not reach statistical significance (F(1, 28) = 3.18, *p* = 0.085, ηp^2^ = 0.102), indicating that the magnitude of change over time did not differ significantly between groups. Despite the small-to-moderate interaction effect size, the lack of statistical significance suggests comparable temporal response patterns across groups.

## 4. Discussion

The present findings should be interpreted within the context of a carefully selected baseline population of clinically stable, community-dwelling older men, in whom major confounding influences related to disease status, severe obesity, malnutrition, or dehydration were minimized through strict eligibility criteria. This trial demonstrates that a short-term IMT intervention enhances diaphragm thickness, is associated with changes in hepatic fat–related metrics, and improves functional aerobic capacity in older men. These results are consistent with previous work in elderly women, suggesting that IMT confers robust benefits across sexes [24]. Notably, the observed decline in diaphragm thickness in the control group underscores the risk of age-related deterioration of respiratory muscles in the absence of targeted intervention. The observed changes in liver density are particularly novel. In CT-based assessments, higher liver density values correspond to lower hepatic fat content; thus, the observed increase in density indicates a reduction in fat infiltration rather than an accumulation of fat. Although exercise training is a well-established method for reducing hepatic steatosis, the underlying mechanism remains unclear. There is no well-established physiological pathway linking IMT to hepatic lipid metabolism; therefore, any potential effect on hepatic steatosis should be interpreted as exploratory. Given that the proposed mechanisms linking IMT to liver fat reduction are speculative and lack direct evidence, these findings should be interpreted with considerable caution. Functional capacity outcomes align with prior studies [16,35,36], emphasizing that IMT can translate into improved daily functioning. Quality-of-life analysis revealed modest gains in the physical domain and moderate improvements in the mental domain, suggesting reduced dyspnea, greater independence, and increased psychological confidence. Limitations include a small sample size, a male-only sample, and a short intervention duration. Larger, longer trials, including both sexes and diverse populations, are warranted to validate and extend these findings. Future research should incorporate larger, more diverse cohorts, extended follow-up, and mechanistic biomarkers to clarify the pathways by which IMT exerts systemic effects.

In the present study, the IMT group demonstrated significantly greater increases in diaphragm thickness than the control group, consistent with prior observations indicating age-related reductions in respiratory muscle performance when no targeted training is provided [24]. The increase observed in the IMT group reflects early-phase adaptations commonly observed with resisted inspiratory loading, in which enhanced neural efficiency and improved motor unit recruitment typically precede structural changes. When interpreted in the broader literature, our findings align with prior evidence that IMT can improve respiratory muscle function and diaphragm-related functional outcomes in older adults [37]. However, substantial heterogeneity across IMT protocols has been reported, including differences in total duration, intensity, and session frequency, as highlighted in the meta-analysis by Seixas et al. [2]. Such variability likely contributes to the wide range of effect sizes documented in the literature. In addition, Manifield [38] demonstrated that IMT improves respiratory muscle strength, diaphragm contractility, and exercise capacity in healthy older adults, supporting the physiological plausibility of our results. Kim et al. further reported that respiratory muscle exercise devices can enhance diaphragm thickness in elderly men [37], although differences in measurement methods (e.g., ultrasound vs. CT) limit direct comparison with the present study. Taken together, the available evidence indicates that IMT may enhance diaphragm performance in older adults; nevertheless, the precise mechanisms underlying these improvements—particularly whether the observed gains predominantly reflect neural adaptations rather than true structural hypertrophy—remain to be fully elucidated, especially in light of methodological limitations and heterogeneity across existing training protocols.

Regarding hepatic outcomes, although exercise interventions are consistently recognized as efficacious for reducing hepatic fat content and improving liver health, no direct evidence currently supports the notion that IMT alone significantly alters hepatic fat density. Instead, robust evidence indicates that aerobic, resistance, and combined aerobic–resistance exercise modalities remain the most effective strategies for improving FLD and metabolic health [39,40,41,42,43,44,45,46,47].

Manifield et al. [48] investigated the effects of IMT in older adults and reported that 6MWT values increased in the IMT group, while no changes were observed in the control group. Similarly, Seixas et al. [2] demonstrated a positive trend for IMT in improving inspiratory muscle performance, functional capacity, and exercise tolerance in elderly participants. Albuquerque et al. [32] found that a short IMT program improved functional capacity, as reflected in 6MWT values, among physically active older adults. In another study, Huang et al. [36] reported that IMT significantly improved 6MWT performance in older individuals without chronic obstructive pulmonary disease (COPD), while still improving walking distance among those with COPD. Likewise, Rodrigues et al. [16] observed enhanced 6MWT values in elderly women following IMT.

However, divergent findings have also been reported—for instance, Mills et al. [7] found no effect of IMT on 6MWT outcomes in older adults. The authors attributed the unchanged post-intervention 6MWT distance to high baseline physical performance levels. Such discrepancies relative to the present study may be explained by differences in participants’ initial endurance-training status. Taken together, the available evidence suggests that IMT is a well-tolerated and effective intervention for improving walking capacity and inspiratory muscle strength in older men. The study did not evaluate balance or activity-level parameters; therefore, no conclusions can be drawn in these domains. Although the magnitude of improvement on the 6MWT may vary, the overall body of evidence supports integrating IMT into training programs for older adults.

A substantial number of RCTs and meta-analyses conducted in populations with COPD have shown that IMT significantly improves quality of life, particularly when protocols employ intensities < 60% of MIP, ≥3 sessions per week, and session durations ≤ 20 min [49,50]. In elderly patients with heart failure, IMT protocols conducted at intensities >25% MIP have been reported to improve quality of life, exercise capacity, and dyspnea [51,52]. Evidence from patients with chronic kidney disease further demonstrates that IMT enhances quality of life, respiratory muscle strength, and functional capacity [53]. In patients with pulmonary hypertension, IMT has been shown to improve general health, social activities, and energy levels, although not all quality-of-life domains showed uniform improvement [54]. Moreover, studies in individuals with asthma aged 20–70 years indicate that IMT is positively associated with quality of life [55]. Although the observed changes did not consistently reach statistical significance, the reported effect sizes suggest a potentially meaningful magnitude of change; however, these findings should be interpreted with caution and not considered definitive evidence of a clinically meaningful effect, warranting further investigation.

To the best of our knowledge, very few studies have examined how IMT affects diaphragm morphology, liver fat content, functional capacity, and quality of life in older men. This multidimensional focus represents an essential contribution of the present work. Despite these strengths, several factors should be acknowledged when interpreting the findings. The participant group consisted exclusively of healthy men aged 60–80 years, and the limited sample size restricts the extent to which these results can be generalized. Larger, more diverse cohorts would likely yield findings with greater external validity. The study focused on four primary outcomes—the 6MWT, SF-12 quality-of-life questionnaire, diaphragm thickness, and hepatic fat levels—which provided valuable yet specific insight into the effects of IMT. Another consideration is the short training period (4 weeks), which did not permit assessment of the long-term sustainability of the observed adaptations.

Several limitations of the present study should be acknowledged. First, the absence of participant dropout may reflect the study cohort’s high motivation and the relatively short duration of the intervention; however, this may limit the generalizability of the findings to longer-term or less supervised training programs.

Several baseline factors known to influence functional and metabolic outcomes in older adults—including hydration status, dietary intake, and protein consumption—were not directly quantified in this study. Although participants were instructed to maintain their habitual lifestyle behaviors throughout the intervention period, the lack of objective assessments of these behaviors represents a methodological limitation. Consequently, the findings should be interpreted within the context of a relatively healthy, well-functioning older male population. Therefore, the present findings should not be extrapolated to older adults with clinically significant dehydration, malnutrition, obesity, or chronic disease, in whom baseline physiological characteristics and response profiles may differ substantially.

Assessing hepatic steatosis is a crucial methodological limitation. Liver fat was estimated using a single region-of-interest (ROI) attenuation measurement from non-contrast computed tomography (CT), which is prone to sampling error and influenced by factors such as hepatic iron content, glycogen storage, and fibrosis. Contemporary standards for hepatic fat quantification favor magnetic resonance imaging–proton density fat fraction (MRI-PDFF) or, when MRI is unavailable, controlled attenuation parameter (CAP) assessed by FibroScan. Even when CT is used, validated approaches typically require liver–spleen attenuation ratios or dual-energy CT. Accordingly, the liver fat estimates reported in this study should be interpreted cautiously and regarded as exploratory rather than definitive outcomes.

Similarly, the assessment of diaphragm thickness constitutes a methodological limitation. Measurements were derived from routine clinical CT images obtained during an inspiratory breath-hold at the level of the right kidney’s upper pole. This non-standard approach has lower sensitivity than B-mode ultrasonography, which is considered the gold-standard technique for evaluating diaphragm morphology. Single-slice CT measurements are influenced by factors such as slice thickness, reconstruction parameters, radiation dose, and patient positioning, and inspiratory breath-hold near total lung capacity may artificially increase apparent diaphragm thickness. Consequently, findings regarding diaphragm thickness should be interpreted with caution and considered exploratory.

The IMT protocol itself also represents a limitation. The four-week duration, with a training volume of two sets of 30 breaths per day and intensities progressing from 40% to 50% of MIP, constitutes a relatively low training dose compared with evidence-based protocols, which commonly employ 6–12-week interventions at 50–70% MIP. Previous studies indicate that four weeks of IMT typically yields modest improvements in inspiratory muscle strength (approximately 10–15%), whereas longer interventions can elicit gains of 25–40%. Thus, the present protocol is more likely to capture early neural adaptations rather than maximal strength or hypertrophic responses. Additional limitations include the predominantly home-based nature of the intervention and the limited direct supervision of training sessions. Although technique and intensity were corrected during weekly supervised visits, variability in adherence, load progression, and execution quality cannot be excluded. Furthermore, lifestyle factors such as diet, hydration status, supplement use, and habitual physical activity were not objectively monitored and may have confounded the results. Finally, outcomes related to balance or habitual physical activity were not assessed; therefore, potential effects of IMT on these domains remain unknown. Additionally, the influence of sex-related physiological differences was not evaluated. Future research should employ more extended intervention periods, include both sexes, and incorporate a broader range of physiological and functional parameters to provide a more comprehensive understanding of the long-term benefits of IMT in aging populations.

## 5. Conclusions

This randomized controlled trial found that a four-week IMT intervention resulted in significant increases in diaphragm thickness, liver tissue density, and functional aerobic capacity in older men. Although improvements in the physical component of quality of life were modest, notable gains in mental well-being were observed, suggesting that IMT may also confer psychological and social benefits. Owing to its accessibility, safety, and low implementation cost, IMT appears to be a practical approach for promoting healthier aging in male populations. Further research with larger cohorts and extended follow-up periods is encouraged to validate these outcomes and clarify the underlying physiological pathways.

## Figures and Tables

**Table 1:** Descriptive characteristics of the participants in the control and IMT groups.

Parameters	Control		IMT	
	Mean	S.D.	Mean	S.D.
Age (years)	71.67	7.94	73.27	6.61
Height (cm)	177.07	4.95	176.93	3.45
Weight (kg)	87.60	5.37	82.20	3.65
BMI (kg/m^2^)	27.1	3.23	27.6	3.05
Total physical activity (MET/minute/week)	933.87	462.55	962.23	446.23

## Data Availability

The data supporting the conclusions of this study are publicly available at Figshare (https://doi.org/10.6084/m9.figshare.31073434), in accordance with the CONSORT 2025 checklist requirements [56].

## Supplementary Materials

The following supporting information can be downloaded at: https://www.mdpi.com/article/10.3390/healthcare14020249/s1, All datasets underlying the findings of this article were included in the submitted manuscript and its Supplementary Materials.

## Abbreviations

The following abbreviations are used in this manuscript:

IMT	Inspiratory muscle training
DT	Diaphragm thickness
FLD	Fatty liver density
MIP	Maximal inspiratory pressure
PCS	Physical component summary
MCS	Mental component summary
NAFLD	Non-alcoholic fatty liver disease
6MWT	Six-Minute Walk test
SF-12	12-Item Short Form Health Survey
QoL	Quality of life
